# Modelling the Ozone-Based Treatments for Inactivation of Microorganisms

**DOI:** 10.3390/ijerph14101196

**Published:** 2017-10-09

**Authors:** Agnieszka Joanna Brodowska, Agnieszka Nowak, Alina Kondratiuk-Janyska, Marcin Piątkowski, Krzysztof Śmigielski

**Affiliations:** 1 Institute of General Food Chemistry, Faculty of Biotechnology and Food Sciences, Lodz University of Technology, 90-924 Lodz, Poland; krzysztof.smigielski@p.lodz.pl; 2 Institute of Fermentation Technology and Microbiology, Faculty of Biotechnology and Food Sciences, Lodz University of Technology, 90-924 Lodz, Poland; agnieszka.nowak@p.lodz.pl; 3 Centre of Mathematics and Physics, Lodz University of Technology, 90-924 Lodz, Poland; alina.kondratiuk-janyska@p.lodz.pl; 4 Division of Heat and Mass Transfer, Faculty of Process and Environmental Engineering, Lodz University of Technology, 90-924 Lodz, Poland; marcin.piatkowski@p.lodz.pl

**Keywords:** ozone treatment, inactivation, predictive microbiology, Weibull, microorganisms

## Abstract

The paper presents the development of a model for ozone treatment in a dynamic bed of different microorganisms (*Bacillus subtilis*, *B. cereus*, *B. pumilus*, *Escherichia coli*, *Pseudomonas fluorescens*, *Aspergillus niger*, *Eupenicillium cinnamopurpureum*) on a heterogeneous matrix (juniper berries, cardamom seeds) initially treated with numerous ozone doses during various contact times was studied. Taking into account various microorganism susceptibility to ozone, it was of great importance to develop a sufficiently effective ozone dose to preserve food products using different strains based on the microbial model. For this purpose, we have chosen the Weibull model to describe the survival curves of different microorganisms. Based on the results of microorganism survival modelling after ozone treatment and considering the least susceptible strains to ozone, we selected the critical ones. Among tested strains, those from genus *Bacillus* were recognized as the most critical strains. In particular, *B. subtilis* and *B. pumilus* possessed the highest resistance to ozone treatment because the time needed to achieve the lowest level of its survival was the longest (up to 17.04 min and 16.89 min for *B. pumilus* reduction on juniper berry and cardamom seed matrix, respectively). Ozone treatment allow inactivate microorganisms to achieving lower survival rates by ozone dose (20.0 g O_3_/m^3^ O_2_, with a flow rate of 0.4 L/min) and contact time (up to 20 min). The results demonstrated that a linear correlation between parameters *p* and *k* in Weibull distribution, providing an opportunity to calculate a fitted equation of the process.

## 1. Introduction

The microbiological quality of food products is an important criterion for applying them in the food industry as well as ensuring consumer safety [1]. An interest in microbial inactivation by food researchers has expanded for years [2,3]. So far, numerous nonlinear regression analyses have been proposed for different food preservation technologies. Recently, a rapidly developing subdiscipline has appeared, involving sophisticated mathematical/computer models or equations that together with the prediction of the growth and activity of microorganisms causing food spoilage is regarded as so-called predictive microbiology. Such modeling concerns regarding the multiple growth parameters, along with the selection of those models, provide an effective practical application for predictive microbiology [4].

Among many food preservation techniques that have been applied in the food industry, ozone treatment allows inactivate microorganisms to achieve lower survival rates by minimum ozone doses [5]. This subject has been widely investigated, concerning different kind of models proposed for inactivation of microorganisms. Some reports claim that models for bacterial inactivation using ozone follow first order kinetics [6,7]. On the other hand, van Boekel 2002 [8] studied the inactivation curves of 120 microorganisms, reporting that only 5% of them followed first order kinetics, including deviations such as sigmoid, concave, and convex shapes. Deviations from log-linear curves have been studied by many researchers. For instance, the Chick-Watson model was evaluated to inactivate *Cryptosporidium parvum* oocysts taking into account the minimum ozone dose and contact time needed for sufficient inactivation [9,10,11]. However, this model possesses limited application due to the fact that the microbial elimination rate is not kept at a constant level [12]. Another model proposed for microbial inactivation was the Gompertz equation, which was unsuitable for nonsigmoid survival curves [13,14].

Recently, the Weibull model has drawn more relevant interest from researchers, particularly due to its flexibility, which provides an opportunity to illustrate concave, convex, and linear shapes. Such a model was proposed by Bialka et al. 2008 [15], who concluded its effectiveness in the description of inactivation of *E. coli* O157:H7 and *Salmonella enterica* in strawberries and raspberries after ozone treatment. Similarly, Patil et al. 2010a, 2010b [11,16] have found the Weibull model appropriate for the assessment of *E. coli* ATCC 25922 and NCTC 12900, *Listeria monocytogenes*, and *L. innocua* inactivation in orange and apple juice after ozone exposure. However, taking into account various microorganism susceptibility to ozone that may be dependent on the several factors, including the type of product, the target microorganism, the initial level of contamination, the physiological state of the bacterial cells, the physical state of ozone, and type of an organic material, it is of great importance to develop a sufficiently effective ozone dose to preserve food products using different strains based on the microbial model [17,18].

In view of the above, we designed this study to develop the model for ozone treatment in a dynamic bed of different microorganisms on a heterogeneous matrix, including juniper berries and cardamom seeds. The proposed model may be useful to optimize inactivation treatments by ozone exposure.

## 2. Materials and Methods

### 2.1. Plant Material Matrix

Two heterogeneous matrices, used in the presented study, consisted of various kinds of spices: juniper berries and cardamom seeds, depending on the diversity of the surface structure. Juniper (*Juniperus communis* L.) berries were collected in the north-eastern region of Poland—Podlaskie province (52.6500° N, 22.7333° E)—and delivered by herbal works KAWON–HURT in Wielkopolska, Poland [19]. Cardamom (*Elettaria cardamomum* (L.) Maton) seeds were obtained from a local store and are native to Guatemala (15.494008° N, 90.798511° W).

### 2.2. Microorganisms

Five bacterial (*B. subtilis, B. pumilus, B. cereus, E. coli,* and *P. fluorescens*) and two fungal strains (*A. niger* and *E. cinnamopurpureum*) were investigated. *Bacillus* strains and *E. cinnamopurpureum* were previously isolated from spices. Bacterial strains were identified based on 16S rRNA gene sequencing, and the nucleotide sequences were deposited in the NCBI GenBank database with accession numbers KP676166 (*B. subtilis* 2J), KP676167 (*B. pumilus* 3J), and KP676168 (*B. cereus* 4J). *E. cinnamopurpureum* was identified by morphological traits [20]. Other microorganisms were originated from culture collection: ATCC (*E. coli* ATCC 10536, *P. fluorescens* ATCC 51821) and ŁOCK (*A. niger* ŁOCK 0431).

### 2.3. Ozone Treatment in Dynamic Bed

**Figure 1:**
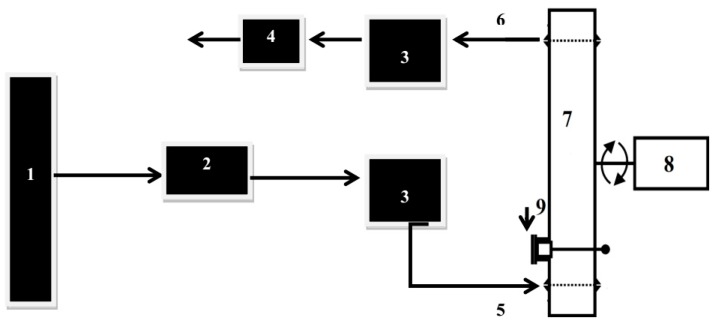
Ozone treatment system in dynamic bed for gaseous phase used for laboratory purposes; 1—oxygen bottle, 2—ozone generator, 3—ozone analyzer, 4—surplus gas elimination unit, 5—inlet of ozone, 6—outlet of ozone, 7—reactor, 8—control system with jolting and rotating mechanism, 9—supply and disposal of plant material treated with ozone [22].

The procedure of ozone treatment in a dynamic bed (gaseous phase) was performed in a laboratory system consisting of a few basic components such as the gas (pure oxygen), the ozone generator, the electric power source, the reactor (cylindrical glass and steel chambers) directly connected to the control system with a jolting and rotating mechanism, the surplus gas elimination unit, and the ozone analyzer (Figure 1) [21,22]. Ozone, previously generated from an oxygen bottle by a laboratory Ozone Generator BMT 803 N (BMT Messtechnik, Berlin, Germany), was transferred to a 1-L reactor in which was placed the contaminated sample of 0.025 L of cardamom seeds and 0.030 L of juniper berries (10.0 g each). The control system with the jolting and rotating mechanism allowed us to operate the ozone treatment process. The ozone analyzer BMT 964 (BMT Messtechnik, Berlin, Germany) allow us to determine ozone concentrations both at the inlet and outlet [20]. The ozone treatment was carried out as follows: inoculated samples were treated with different ozone doses (5.0, 10.0, 15.0, 20.0 g O_3_/m^3^ O_2_) stimulated with different flow rate values (1.2, 0.8, 0.6, 0.4 L/min, respectively) during different contact times (5, 10, 20, 30, 40 min), which gave 80 different measurement conditions. The pressure was kept constant at 0.5 atm. After treatment, each sample was transferred to sterile packaging (Merck, Darmstadt, Germany). Moreover, an ozone sensor (Eco Sensors Model A-21ZX, Newark, NJ, USA) was installed to be able to keep track of ozone concentration in the laboratory.

### 2.4. Inoculation of Plant Material

Before inoculation of the plant material, the bacterial strains were activated on TSA medium (Merck, Darmstadt, Germany) (30 °C, 48 h) and fungal strains on YGC medium (Merck, Darmstadt, Germany) (25 °C, 72 h). After activation, suspensions of the investigated strains were prepared. Bacterial suspensions (2 in McFarland scale, 6 × 10^8^ cfu/mL) were done in a peptone salt solution (Sigma-Aldrich, St. Louis, MO, USA), and fungal suspensions (10^7^ conidia per mL) were done in pepton salt with Tween 80 (Sigma-Aldrich, St. Louis, MO, USA) (1%, *v/v*). Tween 80 minimizes clumping of spores and improves the accuracy of spore counts [23]. Thermally sterilized (121 °C, 15 min) samples of plant material (10 g) were inoculated with 0.2 mL of the appropriate microorganism suspensions in the sterile bags (ChemLand, Stargard, Poland). The samples were mixed to facilitate adherence of the strains to the plant material and were left for 1 h prior to ozone treatment. Inoculated samples were placed into the reactor chamber and treated with ozone. Before and after the decontamination process, the number of bacteria (PCA medium (Merck, Darmstadt, Germany), 30 °C, 72 h) and fungi (YGC medium, 25 °C, 120 h) was determined. The obtained results were expressed as log cfu per gram of sample. All experiments were carried out in triplicate.

### 2.5. Mathematical Model

The modelling was carried out into two stages. First, primary modelling included the fitting of survival curves with the Weibull model. The model was suggested by Peleg and Cole (1998) [24], who concluded the following Equation (1):

(1) $$\ln(N/N_{0})=-{kt}^{p}$$

where *N* means the bacterial concentration at time *t*, *N_0_* is its initial value at time 0, *k* is the scale parameter of the model, *p* is the shape parameter of the model, and *t* is the time of exposure to ozone. Such equation can demonstrate different shapes of survival curves depending on the *p* value. Thus, there may be cases showing a concave downward survival curve if *p* > 1, which informs us that the hazard increases, or a convex shape of the survival curve if *p* < 1, reporting that the hazard is constant, and a linear function if *p* = 1, which means that the hazard decreases [2,25]. Additionally, shape parameter can be described as an indicator of the resistance of the microbial population. Also, knowing the values of scale and shape parameter, the reliable life *t_R_* can be calculated, or the 90% of the failure time distribution, which is analogous to the *D*-value (egn 2) [15].

(2) $$t_{R}=\frac{1}{\sqrt[{p}]{k\times \log e}}$$

In the second step, polynomial equations were used to describe the effects of ozone concentration and incubation time on the parameters *k* and *p* of the Weibull model. Due to the fact that the parameters *k* and *p* are strongly correlated, we decided to model only the shape parameter *p* and then estimate the parameter *k* from the observed strong correlation. We have chosen this parameter because the shape parameter was more affected by the measurement errors of the data (for instance, the sampling errors in the log cell concentrations), thus it was important for research to minimize the error on *p*.

Based on the experimental data the parameters *p* and *k* were estimated using the method of least squares in a modified dependency (1) to the following linear form (3) [26]:

(3) $$\ln\left(-\ln\left(N/N_{0}\right)\right)=\ln k+p\ln t$$

All experiments were carried out in triplicate. To achieve a best-fit, parameter estimation was performed by minimizing the sum of square error between the experimental and estimated log10 reductions, using linear least squares regression method (LINEST function) in Microsoft Excel 2000 (Microsoft, Redmond, WA, USA). Validation of the model was conducted by back-predicting experimental data set and performing a linear regression with the estimated versus the experimental data.

## 3. Results and Discussion

The reduction of *B. cereus*, *B. subtilis*, *B. pumilus*, *E. coli*, *P. fluorescens*, *E. cinnamopurpureum* and *A. niger* after treatment with gaseous ozone in a dynamic bed was evaluated. Based on the results of microorganism survival modelling after ozone treatment and considering the least susceptible strains to ozone, we have chosen the critical ones. Strains from genus *Bacillus*, in particular *B. subtilis* and *B. pumilus*, possessed the highest resistance to ozone treatment because the time needed to achieve the lowest level of its survival was the longest (up to 17.04 min and 16.89 min for *B. pumilus* reduction on the juniper berry and cardamom seed matrix, respectively) (Table 1 and Table 2). Additionally, these two strainsm with phenotypical and genotypical similarity to each other, and related species of *Bacillus* group are widely distributed in the environment and therefore can contaminate all types of food and its products via dust [27]. It was reported that *B. pumilus* is implicated in incidents of food-borne illness. From et al. 2007 [28] demonstrated that pumilacidin produced during *B. pumilus* growth is responsible for food poisoning. However, concerning those aerobic bacteria that form endospores, *B. cereus* attracts the most attention due to the commonness of occurrence in products of plant origin as a foodborne pathogen. It may be found in several types of food, including cereals, spices, milk or its products, egg products, and meat, causing food spoilage, food-borne infections, and intoxication. Furthermore, *B. cereus*, depending on the stage of its growth, is responsible for two different types of food poisoning: emetic and diarrhoeal. The first one is caused by *B. cereus* spores, with nausea and vomiting as the major symptoms. The second type of food poisoning is revealed by abdominal pain and diarrhea, which is caused by heat-labile enterotoxins formed during vegetative growth of *B. cereus* in small intestine. Due to the fact that we have recognized the genus *Bacillus* as the most critical group of microorganisms in the presented study, this study takes into account that *B. cereus* is one of the microorganisms with the most spoilage in the plant material environment [27,28,29,30].

All ozone treatments exhibit a similar trend—the tailing off of survivor strains. The first approach taken to model the data was Weibull model. Three sets of data were fit using linear regression to determine the correlation coefficient (R^2^), and the root mean square error (RMSE) was used as a measure of goodness-of-fit (Table 1 and Table 2).

For the *B. cereus* Weibull model, the RMSE values were 0.01, 0.02, 0.02, and 0.01 for juniper berries treated with ozone doses of 5.0, 10.0, 15.0, and 20.0 g O_3_/m^3^ O_2_ with the flow rate of 1.2, 0.8, 0.6, and 0.4 L/min, respectively, and for the cardamom seed matrix, the RMSE values were 0.04, 0.01, 0.03 and 0.02 for ozone doses of 5.0, 10.0, 15.0, and 20.0 g O_3_/m^3^ O_2_ with the flow rate of 1.2, 0.8, 0.6, and 0.4 L/min, respectively. Concerning the Weibull model inactivation of *B. subtilis* inoculated on the juniper berry and cardamom seed matrix, it can be observed that the failure of the model to accurately estimate log values was higher than in *B. cereus* model reaching 0.20, 0.22, 0.05, and 0.03 RMSE values for juniper berries and 0.05, 0.18, 0.21, and 0.22 for cardamom seeds treated with ozone doses of 5.0, 10.0, 15.0, and 20.0 g O_3_/m^3^ O_2_ with the flow rate of 1.2, 0.8, 0.6, and 0.4 L/min, respectively. RMSE values for *B. pumilus* Weibull model were recorded at the same level as *B. subtilis*, but slightly lower values were observed for juniper berry matrix.

Taking into account the correlation coefficient values of *B. subtilis* (0.73, 0.73, 0.92, and 0.98 for juniper berries as well as 0.99, 0.86, 0.82, and 0.81 for cardamom seeds treated with ozone doses of 5.0, 10.0, 15.0, and 20.0 g O_3_/m^3^ O_2_ with the flow rate of 1.2, 0.8, 0.6, and 0.4 L/min, respectively) and *B. pumilus* (0.91, 0.84, 0.89, and 0.92 for juniper berries and 0.99, 0.91, 0.84, and 0.93 for cardamom seeds treated with ozone doses of 5.0, 10.0, 15.0, and 20.0 g O_3_/m^3^ O_2_ with the flow rate of 1.2, 0.8, 0.6, and 0.4 L/min, respectively) in Weibull model, most of them varied among the ozone parameters, but were at acceptable range (R^2^ = 0.80–0.99) for juniper berry and cardamom seed matrix (Table 1 and Table 2). The Weibull model of microbial inactivation of *E. coli* and *P. fluorescens* demonstrated similar fitting parameters as *B. cereus* comparing RMSE and R^2^ values. Concerning the Weibull model for *E. cinnamopurpureum* and *A. niger* inactivation, it can be concluded that it has a comparable goodness-of-fit of Weibull model as *B. cereus* as well as gram-negative bacteria (*E. coli* and *P. fluorescens*) (Table 1 and Table 2).

**Figure 2:**
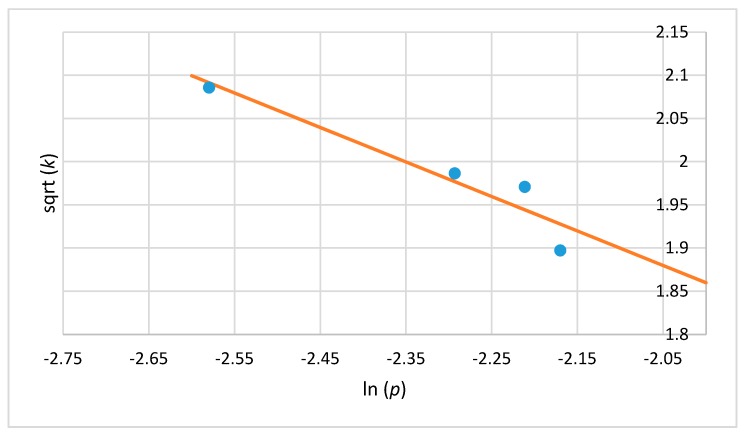
Linear relationship between ln (*p*) and sqrt (*k*) for *B. cereus* on the juniper berry matrix, where *p* and *k* are the scale and shape parameters of the Weibull model (Equation (4)).

**Figure 3:**
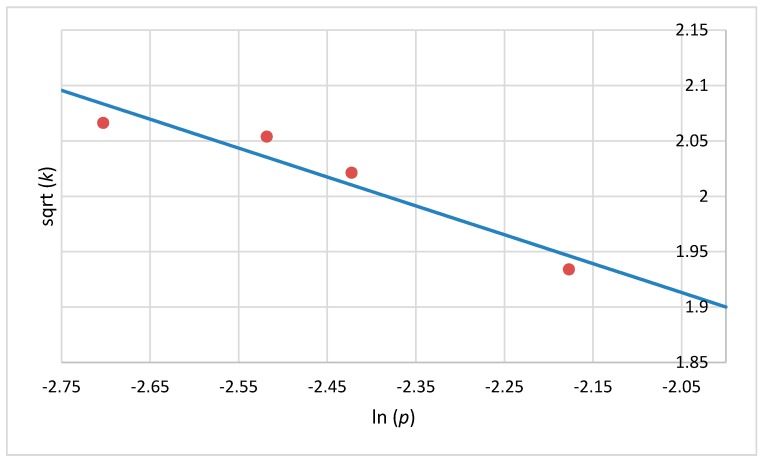
Linear relationship between ln (*p*) and sqrt (*k*) for *B. cereus* on the cardamom seed matrix, where *p* and *k* are the scale and shape parameters of the Weibull model (Equation (5)).

The Weibull model is successfully described by two parameters: *p* and *k* (Table 1 and Table 2). As with the reductions obtained after ozone treatment in a dynamic bed, the shape parameter values (*p*) were less than 1, which indicates that the majority of the remaining cells are resistant to the ozone and therefore have a less probability of dying. The second scale parameter (*k*) corresponds to the mean of the distribution describing death times, thus a failure time or the reliable time *t_R_* in which a 1-log reduction occurs may be determined. According to the *t_R_* values, the susceptibility of microorganisms was in the following order: *A. niger* > *E. cinnamopurpureum* > *P. fluorescens* > *B. cereus* > *E. coli* > *B. subtilis* > *B. pumilus* and *A. niger* > *E. cinnamopurpureum* > *P. fluorescens* > *E. coli* > *B. cereus* > *B. pumilus* > *B. subtilis* for juniper berry and cardamom seed matrix, respectively. Comparing two kind of matrices, the reductions of *A. niger* and *E. cinnamopurpureum* occurred the fastest among the studied strains on both juniper berries and cardamom seeds, with slightly lower *t_R_* values of 3 × 10^−9^ min, 8 × 10^−10^ min, 1 × 10^−4^ min, and 6 × 10^−7^ min for cardamom seeds, whereas *t_R_* values for juniper berries were 6 × 10^−5^ min, 9 × 10^−6^ min, 4 × 10^−4^ min, and 7 × 10^−6^ min. In addition, it can be observed that *k* increases with decreasing *p* and suggests a good correlation between the two parameters. This is due to a monotonic correlation between parameters *p* and *k,* which makes it possible to calculate a fitted equation of the process. Our observations were confirmed by Figure 2 and Figure 3, which clearly show a linear correlation between ln (*p*) and sqrt (*k*) for *B. cereus* on the juniper berry and cardamom seed matrix. These results agree with the work conducted by Couvert et al. 2005 [31], who evaluated a structural correlation between *p* and *k* in their study on the thermal resistance of *B. pumilus*. Similarly, Le Marc et al. 2009 [2] developed the model for the photo-destruction of the foodborne pathogen *B. cereus*, initially treated with a precursor of endogenous photosensitizers, reporting a strong correlation between parameters *k* and *p*. The final equation was estimated for *Bacillus* strains as critical strains inoculated on juniper berry and cardamom seed matrix.

Sqrt (*k*) was modelled using the linear correlation between ln (*p*) and sqrt (*k*). The fitted equations for *B. cereus* (R^2^ = 0.90, R^2^ = 0.92 for juniper berry and cardamom seed matrix, respectively, Equations (4) and (5)), *B. subtilis* (R^2^ = 0.90, R^2^ = 0.92 for juniper berry and cardamom seed matrix, respectively, Equations (6) and (7)) and *B. pumilus* inactivation (R^2^ = 0.98, R^2^ = 0.98 for juniper berry and cardamom seed matrix, respectively, Equations (8 ) and (9)) were written as:

Sqrt (*k*) = 1.062 − 0.3993 × ln (*p*) (4)

Sqrt (*k*) = 1.378 − 0.26073 × ln (*p*) (5)

Sqrt (*k*) = 0.607 − 0.445 × ln (*p*) (6)

Sqrt (*k*) = 0.165 − 1.429 × ln (*p*) (7)

Sqrt (*k*) = 0.408 − 0.5731 × ln (*p*) (8)

Sqrt (*k*) = 0.316 − 0.860 × ln (*p*) (9)

**Figure 4:**
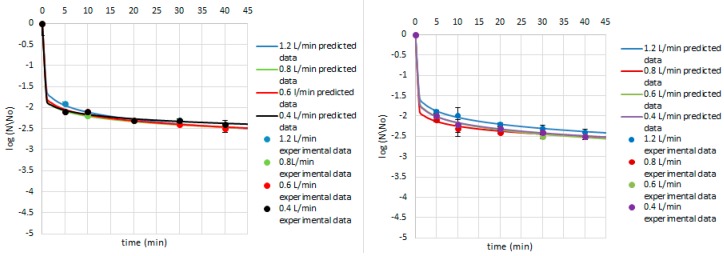
Survival of *B. cereus* on the cardamom seed (**left**) and juniper berry (**right**) matrix treated with ozone doses stimulated with flow rate of 1.2, 0.8, 0.6 and 0.4 L/min after 5, 10, 20, 30, and 40 min. Solid line: prediction of the Weibull model (egns 4–5), dotted line: bacterial log counts.

**Figure 5:**
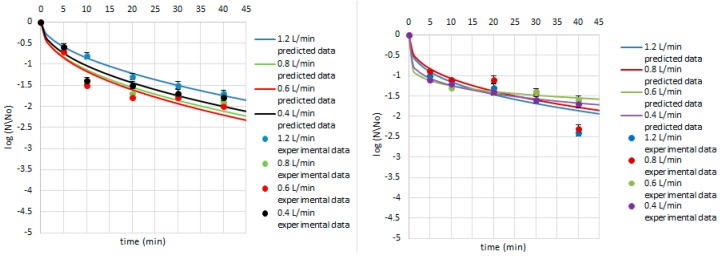
Survival of *B. subtilis* on the cardamom seed (**left**) and juniper berry (**right**) matrix treated with ozone doses stimulated with flow rate of 1.2, 0.8, 0.6, and 0.4 L/min after 5, 10, 20, 30, and 40 min. Solid line: prediction of the Weibull model (egns 6–7), dotted line: bacterial log counts.

**Figure 6:**
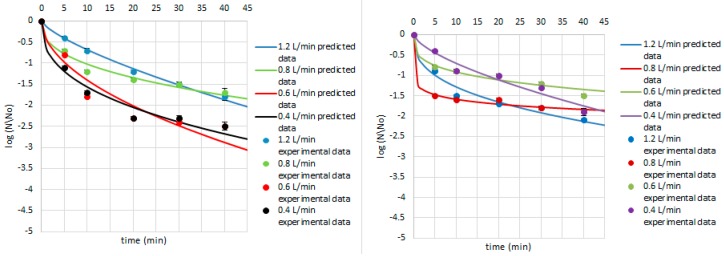
Survival of *B. pumilus* on the cardamom seed (**left**) and juniper berry (**right**) matrix treated with ozone doses stimulated with flow rate of 1.2, 0.8, 0.6, and 0.4 L/min after 5, 10, 20, 30, and 40 min. Solid line: prediction of the Weibull model (egns 8–9), dotted line: bacterial log counts.

Equations (4)–(9) were used to predict the survival curves for the experimental conditions studied in this work. Comparison between the predicted and experimental survival curves shows that the model usually provides a good overall description of the data (Figure 4, Figure 5 and Figure 6). Our study showed that by modelling the data by Weibull, it is possible to predict which strains were critical for the process and indicate how the parameters of ozone treatment should be designed. Therefore, the first step of ozone treatment should be followed by selection of critical strain or strains for each kind of contaminated product. Furthermore, predicted data show that time of the process needed to achieve the lowest survival rate of critical microorganisms did not exceed 20 min. Thus, ozone treatment is considered to be a cost-effective and eco-friendly food processing technology. Ozone technology can be beneficial due to the fact that there are the lower costs of the purchase and maintenance of the ozone supply units compared to the cost of the purveyance of disinfectants [22]. However, it should be taken into account that the efficiency of the ozone treatment may vary depending on the reactor volume, the mass of sample treated with ozone, or the flow rate value [15].

**Figure 7:**
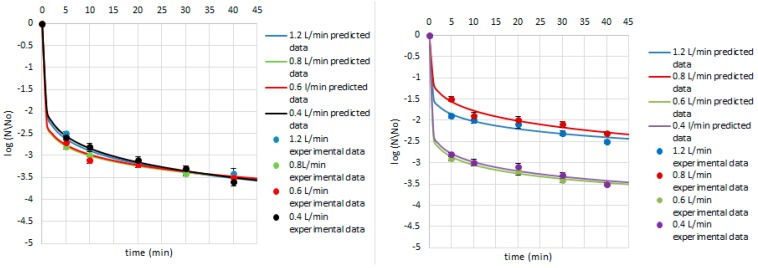
Survival of *E. coli* on the cardamom seed (**left**) and juniper berry (**right**) matrix treated with ozone doses stimulated with flow rate of 1.2, 0.8, 0.6, and 0.4 L/min after 5, 10, 20, 30, and 40 min. Solid line: fit of the Weibull model (egn 1), dotted line: bacterial log counts.

**Figure 8:**
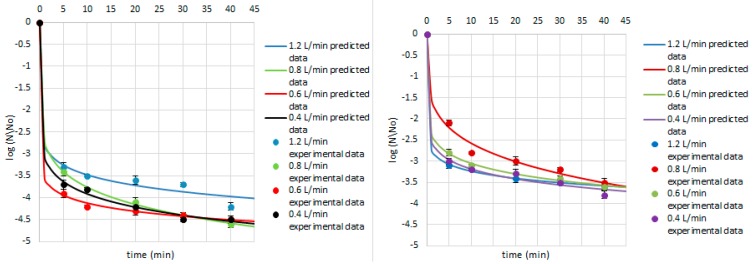
Survival of *P. fluorescens* on the cardamom seed (**left**) and juniper berry (**right**) matrix treated with ozone doses stimulated with flow rate of 1.2, 0.8, 0.6, and 0.4 L/min after 5, 10, 20, 30, and 40 min. Solid line: fit of the Weibull model (egn 1), dotted line: bacterial log counts.

**Figure 9:**
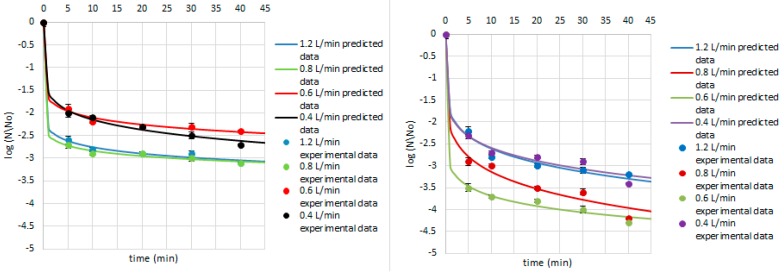
Survival of *E. cinnamopurpureum* on the cardamom seed (**left**) and juniper berry (**right**) matrix treated with ozone doses stimulated with flow rate of 1.2, 0.8, 0.6, and 0.4 L/min after 5, 10, 20, 30, and 40 min. Solid line: fit of the Weibull model (egn 1), dotted line: bacterial log counts.

**Figure 10:**
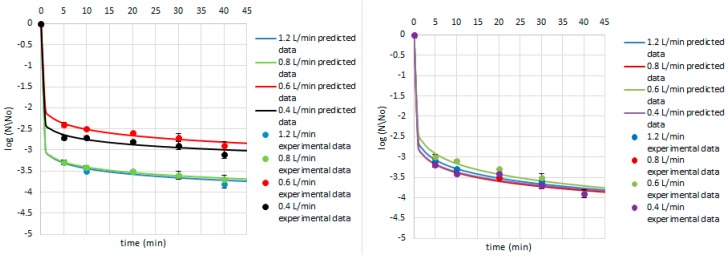
Survival of *A. niger* on the cardamom seed (**left**) and juniper berry (**right**) matrix treated with ozone doses stimulated with flow rate of 1.2, 0.8, 0.6, and 0.4 L/min after 5, 10, 20, 30, and 40 min. Solid line: fit of the Weibull model (egn 1), dotted line: bacterial log counts.

Concerning the Weibull model, it can be concluded that it was appropriate to describe a non-linear inactivation curves (Figure 4, Figure 5, Figure 6, Figure 7, Figure 8, Figure 9 and Figure 10). Such findings are in agreement with Bialka 2008 [15] who developed the Weibull model of *E. coli* O157:H7 and *Salmonella* on blueberries, raspberries, and strawberries after ozone or pulsed UV-light treatment, indicating a non-linear character of survival curves. Therefore, the Weibull model can be a useful tool to exhibit the appropriate shape of survival curves. The fit of the Weibull model to the experimental data can be seen in Figure 7, Figure 8, Figure 9 and Figure 10. The survival curves of ozone treatment may display different shapes such as convex, concave, and linear shapes [2]. Our results indicated that all cases taken into account in this study showed the situation where *p* value is 0 < *p* < 1, referring to the convex shape of survival curves (Figure 7, Figure 8, Figure 9 and Figure 10) possessing the monotonically decreasing character of both density and intensity.

Following ozone treatment, the survival rate of microorganisms decreased sharply (Figure 4, Figure 5, Figure 6, Figure 7, Figure 8, Figure 9 and Figure 10) on both the juniper berry and cardamom seed matrix. A clear dependence of ozone treatment efficiency on ozone contact time and ozone dose was observed. The conducted study indicated that increasing ozone concentration and contact time increases the decay rates at which the sensitive cells are killed in the first minutes of ozone treatment. However, the cells that survive become more resistant to ozone. That was confirmed in our previous observations, concluding that the effectiveness of ozone treatment decreases with time. It means that a longer ozone contact time may have a negative impact on the product quality from the microbiological perspective, causing its further resistance to ozone and reactivation. This finding agreed with the previous data from Brodowska et al. 2014, 2015 [20,29], which proved that a prolonged period of ozone treatment was not effective in microbial inactivation. Such problems with selecting the proper parameters may be resolved by mathematical modelling.

Secondary modelling focuses on the effect of the environmental factors on the main parameters of the primary model. Due to the fact that the shape parameter *p* varies greatly with the experimental conditions, it was impossible to use a single *p*-value for the whole data set. Therefore, we modelled only the shape parameter and then estimated the second-scale parameter from their correlation. The best results were obtained by model sqrt (*k*) as a function of ln (*p*).

Taking into account the experimental data among Gram-positive bacteria, *B. cereus* was indicated as the most sensitive to ozone no matter which matrix was used, and there was an almost 2-log reduction after a 5-min treatment with the lowest ozone dose (5.0 g O_3_/m^3^ O_2_) (Figure 4). Concerning other conditions, namely lower ozone concentrations and longer times of exposure, similar reductions were obtained as previous described. Our results are in disagreement with those obtained by Akbas and Ozdemir 2008 [32], who reported that ozone concentrations of 0.0002 and 0.001 g O_3_/m^3^ O_2_ after a 360-min treatment resulted in 2.7 and 2.9-log reductions in population of *B. cereus*, respectively. The same authors also noticed that using higher ozone doses, such as 0.011, 0.015, and 0.019 g O_3_/m^3^ O_2_, were needed to achieve significant reductions in *B. cereus* spores in dried fig samples, 1.5, 2.0, and 2.0-log reductions were observed, respectively [32]. In another study the same authors found ozone to be effective in inactivation *B. cereus* in pistachios, reporting that the effectiveness of ozone increased with increasing exposure time and ozone dose. At low ozone concentrations (0.0002 and 0.001 g O_3_/m^3^ O_2_, 360 min), *B. cereus* counts were decreased by 1.5–2.0 log numbers [33].

Taking into account *B. subtilis* inactivation, it can be concluded that treated cardamom seeds and juniper berries with ozone met with resistance with respect to a 20-min treatment (Figure 5). It was reduced by less than 2 and 1.5 log numbers in the whole ozone concentration range on the cardamom seed and juniper berry matrix, respectively. Comparing the sensitivity of *B. subtilis* to ozone in cardamom seeds with juniper berries, there was no significant reduction after a 5-min treatment (Figure 5), whereas the same time was sufficient for *B. cereus* inactivation and was reduced by 2 log numbers in both cardamom seed and juniper berry samples. Our observations disagree with those evaluated by Akbas and Ozdemir 2006 [33], who verified that *B. cereus* counts were decreased by 2 log numbers in ground pistachios after ozone treatment at a dose of 0.002 g O_3_/m^3^ O_2_ for 360 min, whereas 3.5-log reduction was recorded in kernels and shelled pistachios under the same processing conditions. It means that in their study 6 h and a much lower ozone concentration (1000 times) were needed for achieving the same level of reduction of *B. cereus*, while in our study 5 min with using higher ozone dose was sufficient. Undeniably, the longer time of the process is associated with a higher cost for whole process, whereas the aim of development of each new technology is to reduce the cost of the process as much as possible.

Similarly to *B. subtilis*, *B. pumilus* (Figure 5 and Figure 6) was not significantly affected with ozone at its lowest doses as *B. cereus*, and the exposure time required elongation up to 20 min using juniper berries or cardamom seeds as a matrix. During this time, about a 2.0-log reduction was achieved in juniper berry samples treated with ozone doses of 5.0 and 10.0 g O_3_/m^3^ O_2_ with the flow rate of 1.2 and 0.8 L/min, respectively, whereas a similar level of reduction in matrix consisted of cardamom seeds was obtained for higher ozone doses of 15.0 and 20.0 g O_3_/m^3^ O_2_ with the flow rate of 0.6 and 0.4 L/min, respectively.

On the other hand, Gram-negative bacteria demonstrated a greater sensitivity to ozone than Gram-positive ones (Figure 7 and Figure 8). Concerning the impact of ozone treatment on *P. fluorescens* survival, it was evaluated that the lower survival rate indicated samples of cardamom seeds, revealing a 3.2–4.0 log decrease during a 5-min treatment over the range of ozone doses. *B. cereus* population was more resistant to ozone than *P. fluorescens* ones because the survival of its counts in cardamom seed samples was two orders higher than in *P. fluorescens*. Additionally, it was observed that *P. fluorescens* counts in juniper berries were less reduced than in cardamom seed matrix, a 2.1–3.1 log decrease in all ozone concentrations after the shortest period of treatment was reported (Figure 8). During treatment of samples contaminated with *B. cereus*, it can be noticed that the matrix does not have an impact on the survival of *B. cereus*. Such differentiation in inactivation of *P. fluorescens* occurred in the cardamom seed and juniper berry samples may be due to the diversity of their surface structure. Juniper berries are characterized by a smooth and shiny surface structure, while cardamom seeds possess a rough, rather dry appearance. Therefore, it was confirmed that the surface area is an important factor for evaluation the effectiveness of ozone treatment. This may be explained by some kind of protection in ground pistachios from oxidant effect of ozone. The authors suggested that the formation of such a barrier can be caused by the reduction of ozone penetration and interaction with organic components, including lipids and carbohydrates, present in pistachios [33]. Also, Naitoh et al. 1989 [34] and Zagon et al. 1992 [35] found that cereal flour and ground pepper treated with ozone required a higher ozone dose and a prolonged period of treatment than the whole cereal and pepper to obtain the same level of microbial reduction. Additionally, the differences in the product treated with ozone are attributed to its surface structure and are also related to the content of biologically active compounds (polyphenols, essential oils) and antioxidant activity [29].

Concerning *E. coli* inactivation after ozone treatment, it was concluded that the strain demonstrated about 3.0-log reduction for higher ozone doses (15.0 and 20.0 g O_3_/m^3^ O_2_ with the flow rate of 0.6 and 0.4 L/min, respectively) after a 5-min treatment on juniper berry matrix, whereas the same results were achieved on cardamom seed matrix over the whole ozone concentration range during the same time.

Our results are in disagreement with the findings of Restaino et al. 1995 [17], which concluded that Gram-positive bacteria such as *L. monocytogenes* was more sensitive to aqueous ozone than Gram-negative ones (*E. coli*, *Yersinia enterocolitica*). Kim et al. 1999 [36] and Victorin 1992 [37] found Gram-positive bacteria as more resistant than Gram-negative ones due to the higher amount of peptidoglycan in their cell walls. This is due to the fact that primary destruction of the lipoprotein and lipopolysaccharide layers occurs in Gram-negative bacteria, an increase in the cell permeability and eventual cell lysis is noticed [36,37,38].

Additionally, the presented study included the assessment of the fungal ability to survive during ozone exposure. Samples of juniper berries treated with ozone doses of 10.0 and 15.0 g O_3_/m^3^ O_2_ stimulated with the flow rate of 0.8 and 0.6 L/min, respectively, after 5 min, achieved a 2.9 and 3.5 log decrease in *E. cinnamopurpureum* counts, respectively, whereas treatment of cardamom seeds under the same conditions resulted in a lower reduction—2.7 and 1.9 log numbers, respectively (Figure 9). The obtained results showed that *E. cinnamopurpureum* is more sensitive to ozone than *B. cereus*.

Taking into account the effect of ozone treatment on the *A. niger* inactivation, we concluded that the lowest ozone concentrations in both juniper berry and cardamom seed samples demonstrated similar level of decontamination up to 10 min, while with increasing period of ozone treatment, the lower survival rate was achieved in juniper berry samples (Figure 10). Comparing the inactivation of *B. cereus* to *A. niger* by ozone, we concluded that the second one achieved twice the reduction after treatment.

The conducted study show that two tested fungal species possessed different sensitivity to ozone. This observation agreed with previous data from Palou et al. 2001 [39], who compared the impact of ozone on *Penicillium italicum* and *P. digitatum* growth and proved that the first one was affected by ozone, while *P. digitatum* was found to be resistant. What is more, Miller et al. 2013 [18] recognized gaseous ozone as the most relevant to inactivate fungi.

## 4. Conclusions

All data presented in this study clearly revealed that ozone-based treatment can be an effective and suitable choice for microorganism inactivation. A good correlation between the Weibull model parameters *k* and *p* allow us to obtain the final equation describing parameters of ozone treatment. Therefore, the Weibull model may be a useful tool to select a sufficiently effective ozone dose and contact time for contaminated plant material. Taking into account that the efficacy of ozone on microbial inactivation depends on the various factors, such as a type of the product, target microorganism, initial level of contamination, physiological state of the bacterial cells, the physical state of ozone, and the type of organic material, it is hard to optimize ozone processing conditions. Thus, those should be assessed for a particular product, once the microbial quality can be affected. In addition, our study showed that by modelling the data by Weibull, it is possible to predict which strains were critical for the process and indicate how the parameters of ozone treatment should be designed. Therefore, the first step of ozone treatment should be followed by the selection of the critical strain or strains for each kind of contaminated product. Additionally, prior to the design of ozone treatment, it should be taken into consideration how the efficiency of the ozone treatment may vary depending on the reactor volume, the mass of sample treated with ozone, or the flow rate value.

Nevertheless, this paper established that ozone treatment possesses a promising potential as an eco-friendly decontamination method, but further work is needed to develop the applicability of the model and the effectiveness of the ozone-based treatment from a food industry perspective. Therefore, further goals will mainly be focused on how the model will works in reality, ensuring a low survival rate of microorganisms in food products without having an unfavorable effect on its visual, textural, and nutritional quality.

## Figures and Tables

**Table 1:** Weibull model and fitted parameters for reductions of *B. cereus*, *B. subtilis*, *B. pumilus*, *E. coli*, *P. fluorescens*, *E. cinnamopurpureum*, and *A. niger* on the juniper berry matrix after ozone treatment in a dynamic bed.

Research Matrix	Microorganism	Ozone Flow Rate (L/min)	Weibull Model Parameters			Fitted Parameters	
			*k*	*p*	*t_R_* (min)	RMSE	R^2^
Juniper berries	*B. cereus*	1.2	3.60	0.11	0.02	0.01	0.99
		0.8	4.35	0.08	0.00	0.02	0.93
		0.6	3.88	0.11	0.01	0.02	0.97
		0.4	3.95	0.10	0.01	0.01	0.98
	*B. subtilis*	1.2	1.20	0.35	6.62	0.20	0.73
		0.8	1.07	0.36	8.25	0.22	0.73
		0.6	2.01	0.16	2.37	0.05	0.92
		0.4	1.74	0.22	3.69	0.03	0.98
	*B. pumilus*	1.2	1.28	0.37	5.03	0.11	0.91
		0.8	2.87	0.11	0.12	0.04	0.84
		0.6	1.12	0.28	13.62	0.10	0.89
		0.4	0.36	0.66	17.04	0.18	0.92
	*E. coli*	1.2	3.50	0.12	0.03	0.04	0.90
		0.8	2.68	0.18	0.44	0.05	0.93
		0.6	5.67	0.09	0.00	0.02	0.97
		0.4	5.46	0.10	0.00	0.02	0.95
	*P. fluorescens*	1.2	6.30	0.07	0.00	0.01	0.99
		0.8	3.56	0.22	0.14	0.06	0.93
		0.6	5.42	0.11	0.00	0.01	0.98
		0.4	5.81	0.10	0.00	0.03	0.91
	*E. cinnamopurpureum*	1.2	4.08	0.17	0.03	0.06	0.90
		0.8	4.91	0.17	0.01	0.06	0.90
		0.6	6.93	0.09	0.00	0.03	0.91
		0.4	4.16	0.16	0.02	0.06	0.88
	*A. niger*	1.2	6.03	0.10	0.00	0.03	0.91
		0.8	6.36	0.09	0.00	0.02	0.95
		0.6	5.58	0.12	0.00	0.04	0.87
		0.4	6.36	0.09	0.00	0.03	0.86

**Table 2:** Weibull model and fitted parameters for reductions of *B. cereus*, *B. subtilis*, *B. pumilus*, *E. coli*, *P. fluorescens*, *E. cinnamopurpureum*, and *A. niger* on the cardamom seed matrix after ozone treatment in a dynamic bed.

Research Matrix	Microorganism	Ozone Flow Rate (L/min)	Weibull Model Parameters			Fitted Parameters	
			*k*	*p*	*t_R_* (min)	RMSE	R^2^
Cardamom seeds	*B. cereus*	1.2	3.74	0.11	0.01	0.04	0.90
		0.8	4.22	0.08	0.00	0.01	0.98
		0.6	4.09	0.09	0.00	0.03	0.91
		0.4	4.27	0.07	0.00	0.02	0.89
	*B. subtilis*	1.2	0.59	0.52	13.75	0.05	0.99
		0.8	0.92	0.45	7.59	0.18	0.86
		0.6	0.94	0.46	7.12	0.21	0.82
		0.4	0.79	0.48	9.34	0.22	0.81
	*B. pumilus*	1.2	0.30	0.73	16.89	0.05	0.99
		0.8	0.96	0.39	9.37	0.12	0.91
		0.6	0.98	0.52	5.18	0.22	0.84
		0.4	1.48	0.39	3.12	0.11	0.93
	*E. coli*	1.2	4.82	0.14	0.00	0.05	0.87
		0.8	5.40	0.11	0.00	0.01	0.99
		0.6	5.32	0.11	0.00	0.03	0.93
		0.4	4.62	0.15	0.00	0.02	0.97
	*P. fluorescens*	1.2	6.44	0.10	0.00	0.05	0.80
		0.8	6.25	0.14	0.00	0.01	0.99
		0.6	8.20	0.06	0.00	0.01	0.96
		0.4	7.06	0.11	0.00	0.02	0.95
	*E. cinnamopurpureum*	1.2	5.37	0.07	0.00	0.02	0.91
		0.8	5.71	0.06	0.00	0.02	0.90
		0.6	3.84	0.10	0.01	0.04	0.88
		0.4	3.57	0.14	0.05	0.03	0.95
	*A. niger*	1.2	6.96	0.06	0.00	0.02	0.86
		0.8	6.95	0.05	0.00	0.01	0.97
		0.6	4.78	0.08	0.00	0.02	0.91
		0.4	5.51	0.06	0.00	0.03	0.78
